# The RESILIENT Dataset: Multimodal Monitoring of Ageing-Related Comorbidities and Cognitive Decline

**DOI:** 10.1038/s41597-025-05958-x

**Published:** 2025-10-22

**Authors:** Nathalia Céspedes Gómez, Yu Chen, Samaneh Kouchaki, Mahan Heydari, Alexandra Cairns, Sergio David Sierra Marín, Alexander Capstick, Jaye Somers, Kirsty Harris, Wilson Wen Bin Goh, Chloe Walsh, Jessica True, Olga Balazikova, Ramin Nilforooshan, Payam Barnaghi

**Affiliations:** 1 Imperial College London, Department of Brain Sciences, London, W12 0NN United Kingdom; 2 The UK Dementia Research Institute, Care Research and Technology Centre, London, United Kingdom; 3 University of Surrey, Guildford, GU2 7XH United Kingdom; 4 Surrey and Borders Partnership NHS Trust, Leatherhead, KT22 7AD United Kingdom; 5 Nanyang Technological University, Singapore, Singapore

**Keywords:** Health care, Biomarkers

## Abstract

The growing ageing population and prevalence of comorbidities pose significant healthcare challenges, from increasing hospitalisations to dementia risk. Healthcare systems primarily treat single conditions, overlooking the complex interplay of chronic diseases. Advances in wearable technology and remote healthcare monitoring technologies offer opportunities to enhance management of comorbidities and early intervention to improve healthcare outcomes. This study presents the RESILIENT dataset, a collection of physiological, sleep, and mental health assessment data conducted as part of an ageing-related comorbidities and dementia study. The RESILIENT study has developed a digital platform to integrate data from wearable devices and in-home monitoring technologies to track physiological, sleep, and cognitive patterns. The validation analysis using the Resilient data highlights correlations between cognitive function, mental health, physical activity, and sleep, aligning with existing literature. By leveraging this dataset, researchers can develop predictive models for early detection and personalised interventions aimed at reducing unplanned hospital admissions and improving health outcomes. The study provides technical foundations and pilot validation for constructing virtual wards to support and complement healthcare services.

## Background & Summary

Globally, long-term conditions are responsible for at least 75% of all deaths, with the majority occurring in low- and middle-income countries^1^. The economic burden of these conditions is substantial, with global costs projected to reach 47 trillion by 2030, posing a serious challenge to healthcare systems worldwide^2^. In the UK, long-term conditions account for up to 70% of annual health and social care spending and 50% of all General Practitioner (GP) appointments, underscoring the significant burden they place on healthcare systems^3^. Transforming care delivery through predictive and preventative strategies has the potential to improve both the quality of services and the quality of life for individuals living with these conditions. Preventative strategies will also reduce the costs associated with the management of long-term conditions. Among long-term conditions, comorbidities such as hypertension, diabetes, cardiovascular disease, and osteoarthritis are commonly linked to cognitive decline and increased frailty in older adults^4–6^. These comorbidities not only exacerbate the progression of cognitive impairment but also complicate clinical management and increase the risk of hospitalisation, care home admission, and mortality^7^. The RESILIENT project^8^ aims to advance personalised healthcare for individuals with long-term conditions by developing a system for continuous, in-home monitoring and analysis of multimodal data. This data is used to develop predictive models for risk assessment in long-term conditions and facilitate the early detection of changes in health status. The predictive models can be used as decision-support and insight-generation tools, enabling timely interventions and improved healthcare outcomes.

A clinically applicable and scalable platform with several essential features is required to support in-home monitoring for individuals with long-term conditions. Such a system must be capable of recording and updating comprehensive individual-level information, including prescribed medications, GP and hospital visits, and co-existing chronic conditions. It should incorporate a user-facing application that enables patients to self-report symptoms and experiences. This would include information that is not readily captured by commercially available in-home sensing technologies, such as dizziness, anxiety, or pain. These requirements were taken into consideration when developing the software framework for the continuous collection of in-home monitoring data as part of the RESILIENT project. This is supported by a data management platform that curates and integrates key information, including demographic data, mental health assessments, physician appointments, and hospital admissions. Importantly, the complete software stack is made publicly available^9^ to lower the cost required to conduct remote healthcare monitoring studies.

The dataset presented in this paper originates from the RESILIENT project. It contains multimodal data collected from 73 older adult participants living with multiple long-term conditions. This includes continuous physiological signals, physical activity, and sleep state data obtained from wearable and remote in-home monitoring devices, along with demographic details and clinically validated mental health assessments such as the Patient Health Questionnaire (PHQ-9), the Generalized Anxiety Disorder scale (GAD-7), the Geriatric Depression Scale (GDS-15) and the Addenbrooke’s Cognitive Examination (ACE-III). The integration of these diverse data sources enables a comprehensive understanding of daily living patterns and health trajectories in ageing populations. This dataset holds significant potential for developing and validating machine learning models for early detection of cognitive and functional decline, predictive risk assessment, and personalised intervention strategies in community healthcare. It can support broader research into the relationships between mental health, comorbidities, and behavioural changes over time.

The contributions presented in this work are:

- We provide a public, open-source repository that contains software to collect, store, and analyse in-home monitoring data: https://github.com/tmi-lab/resilient.
- We make publicly available up to 6 months of in-home physiology, activity, and cognitive assessment data:https://zenodo.org/records/16755408.
- We provide open-source software for aggregating and analysing the dataset, including summary statistics, stratified analyses by gender and age group, and data visualisations: https://github.com/tmi-lab/Resilient-Dataset.
- The platform and validation datasets provide a technical model and pilot data to support constructing virtual wards within primary and secondary healthcare services and for improved in-home monitoring for at-risk groups.

## Methods

The RESILIENT dataset^10^ was collected using an open-source software platform^9^ that integrates data from wearable and remote in-home devices for continuous health monitoring. Data collection involved ethically approved procedures with informed consent from participants, and the final dataset was fully de-identified to ensure privacy and confidentiality.

### RESILIENT platform

The RESILIENT platform^9^ is an open-source digital solution designed to integrate data from remote healthcare monitoring devices, providing comprehensive clinical visualisations and generating insightful reports for both users and healthcare professionals. The system follows a structured architecture to acquire data from Withings wearables^11^, leveraging the official Withings API, which provides access to multiple device-specific measurements.

The platform specifically integrates two Withings devices (Table 1): the ScanWatch and the Sleep Mat. The ScanWatch provides activity and cardiovascular data, including step counts and heart rate readings, each associated with their corresponding timestamps. The Sleep Mat captures detailed sleep-related metrics, including sleep state (with start and end timestamps), as well as sleep physiology parameters such as heart rate, respiration rate, snoring events, and the standard deviation of heart rate calculated over one-minute windows, all timestamped accordingly.

**Table 1:** Summary of data tables and recorded variables.

Device	Table Name	Variables
ScanWatch	**Steps**	- **Steps**: Number of steps recorded per hour.
		- **Timestamp**: Timestamp of each record.
	**Heart Rate**	- **Heart Rate**: Measurements taken every 10 minutes.
		- **Timestamp**: Timestamp of each record.
Sleep Mat	**Sleep States**	- **Sleep State**: One of four sleep states (Light, Deep, REM, Wake up).
		- **Start Timestamp**: Start time of the corresponding sleep state.
		- **End Timestamp**: End time of the corresponding sleep state.
	**Sleep Physiology**	- **Heart Rate**: Measured every minute.
		- **Respiration Rate**: Measured every minute.
		- **Snoring**: Total snoring duration (seconds) per minute.
		- **SDNN 1**: Standard deviation of heart rate over a one-minute window.
		- **Timestamp**: Timestamp of each record.
N/A	**Demographics**	- **Sex**: Male or Female.
		- **Age Group**: One of three age groups: [72–75], [76–87], [88–89].
		- **ACE-III Scores**: Total scores at baseline and 6-month follow-up with individual item scores.
		- **PHQ-9, GDS-15, GAD-7 Scores**: Total scores at baseline with individual item scores.
		- **Assessment Date**: Date each assessment was taken.
		- **Essential Hypertension**: True or False.
		- **Osteoarthritis**: True or False.

**Table 2:** Summary of recording data across variables (*n* = 73).

File	Avg. Days Recorded	Avg. Records/Day	Min Duration (days)	Max Duration (days)
ScanWatch Heart Rate	132.62	102.53	10	184
ScanWatch Steps	138.60	23.80	5	184
Sleep States	136.14	110.50	24	184
Sleep Physiology	106.13	187.84	1	184

**Fig. 1:**
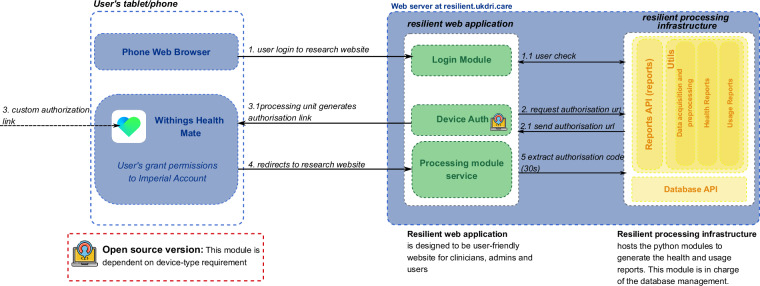
RESILIENT platform architecture: an open-source digital solution designed to integrate data from remote healthcare monitoring. The platform integrates data from Withings wearable devices. The processing infrastructure handles secure data acquisition, storage in a relational database, and the generation of healthcare and usage PDF reports. The web-based application provides healthcare professionals with an intuitive interface for data visualisation, report generation, and raw data access.

As shown in Fig. 1, users authenticate through the Withings Health Mate application^11^, granting permission for data access. The authentication process is managed by the processing infrastructure, which generates an authorisation link and redirects users accordingly to the Resilient web application. Once authorisation is granted, the processing unit extracts the authorisation code, enabling secure data acquisition.

At the core of the Resilient’s processing infrastructure lies a dedicated module responsible for handling healthcare data. This module processes incoming data streams and stores them in a relational database system, which was developed using the Django web framework. The database is structured to support efficient querying, secure data management, and seamless integration with reporting and monitoring services. The stored data is subsequently used to generate detailed PDF reports containing clinically relevant health metrics. In parallel, the infrastructure incorporates a monitoring component that continuously tracks the status of connected wearable devices, including their most recent readings, connectivity status, and battery levels.

Complementing the processing infrastructure, the Resilient platform features a web-based application specifically designed for healthcare professionals. It serves as the primary user interface, enabling users to manage and monitor devices, generate and download reports, and visualise patient health data through interactive dashboards. Additionally, the application offers direct access to raw data, enabling more advanced clinical analyses. Communication between the processing module and web application is managed via secure APIs, ensuring real-time data synchronisation and system responsiveness.

Beyond its core functionalities, the platform is designed to be both adaptable and open-source, allowing for seamless modifications to accommodate specific device requirements. This flexibility ensures compatibility with other wearable technologies while preserving an intuitive and user-friendly experience. In line with the FAIRness considerations outlined in Table 3, the platform supports interoperability at the feature level (e.g., heart rate, sleep stages), enabling integration with data from other devices. Although the current implementation is based on Withings APIs^11^, the underlying data format is structured to allow retrospective harmonisation, and can serve as a template for integrating other wearable APIs used by external researchers. By combining secure data acquisition and visualisation to generate weekly or monthly reports for each individual, the platform serves as a robust tool for remote healthcare monitoring.

**Table 3:** Detailed FAIRness aspects of the RESILIENT dataset.

Aspect	Details
Metadata Availability	A comprehensive data dictionary is provided in the Resilient_dataset_dictionaries.csv file, detailing each file and variable, including data types, units, and descriptions for both device-generated data and demographic information.
Standards for Wearables	While no universal standard exists for wearable data, this dataset follows the format and conventions used by Withings devices and APIs^11^.
Preprocessing	All preprocessing steps are documented and reproducible via open-source scripts provided in the dataset repository https://github.com/tmi-lab/Resilient-Dataset. Specifically, preprocessing is performed in the Preprocessing_data.ipynb notebook, which includes timestamp normalization, daily aggregation of physiological and activity data, and calculation of sleep states from raw sleep mat data.
Interoperability	Harmonisation is possible at the feature level (e.g., heart rate, sleep stages) with other devices. Mapping guidelines should be developed based on the specific devices used by external researchers to ensure compatibility.
API Portability	Raw data is derived from Withings APIs^11^. While not directly portable, the data format can serve as a reference for retrospective harmonisation with other APIs and may guide the integration of data from different wearable device platforms.

### Participants and ethical approval

Participants eligible for the study are individuals aged 65 years or older with a diagnosis of at least two chronic health conditions that increase dementia risk (e.g., arthritis, chronic kidney disease, chronic obstructive pulmonary disease, heart disease or failure, depression or other mental health disorders, diabetes, hypertension, and liver disease, stroke) who may participate with or without a study partner. Demographic characteristics of the study participants, including age, sex, and top comorbidities, are summarised in Table 4. Recruitment was conducted through clinicians at Frailty Hubs, UK National Health Service (NHS) hospitals, and GP surgeries in the southeast of England. Clinicians referred eligible patients or performed database searches and sent invitation letters via post, email, or text message. Interested individuals contacted the research team to register their intent to join the study. Individuals with severe mental health (e.g., severe depression, psychosis, agitation, or anxiety), severe sensory impairment, treatment for a terminal illness at baseline (with a life expectancy of less than six months or being in the last year of life), or an inability to provide informed consent were excluded from the study. All potential participants received a Participant Information Sheet outlining the nature of the study, data usage and retention policies, and their rights under the General Data Protection Regulation (GDPR). They were given at least 24 hours to consider participation and encouraged to consult with family or caregivers before providing written informed consent. Capacity to consent was assessed in accordance with Good Clinical Practice and the Mental Capacity Act 2005. Consent included agreement to participate and to the collection, storage, and sharing of anonymised and pseudonymised data in compliance with NHS and GDPR data protection standards. The principal investigator reviewed individual cases to determine final eligibility.

**Table 4:** Demographics of the participants in the study (*n* = 73).

Sex, *n* = 73	
Female	42
Male	30
Unknown	1

Age, *n* = 73	
72 – 75	11
76 – 87	45
88 – 99	14
Unknown	3

Top Comorbidity, *n* = 73	
Essential hypertension	43
Osteoarthritis	28
Both	24
Neither	8
Unknown	18

The **RESILIENT** study has been reviewed and approved by the London-Surrey Borders Research Ethics Committee and the Health Research Authority and is registered on the Integrated Research Application System (IRAS) under reference number 321104. This publicly available dataset includes remote healthcare monitoring data and baseline mental health and cognitive assessments conducted throughout the monitoring period, providing a comprehensive resource for analysing health trends and detecting early signs of cognitive and physiological decline.

### Dataset Collection

We integrated wearable and in-home sensory data with individuals’ healthcare information extracted from REDCap^12,13^ records to create a comprehensive view of the participants’ well-being and care needs.

Physiological data was acquired through wearable and sleep mat devices, which continuously collected and transmitted data to the RESILIENT platform. Within the platform’s processing infrastructure, data from each device was preprocessed, de-identified, cleaned (removing redundant records), and merged according to the source device. A detailed explanation of the available data is provided in the Data Records.

Mental health, demographics and cognitive assessments for this study were conducted by a monitoring team that contacted participants directly. The dataset includes baseline assessments for ACE-III, PHQ-9, GDS-15, and GAD-7, with 6-month follow-up data available only for ACE-III. The ACE-III is a cognitive screening tool that assesses five domains: attention, memory, fluency, language, and visuospatial function, aiding in the detection of cognitive impairment and dementia^14^. The PHQ-9 is a self-report measure used to assess the severity of depressive symptoms based on the Diagnostic and Statistical Manual of Mental Illnesses (DSM-5 criteria)^15^. The GDS-15 is a short, validated screening tool designed to detect depressive symptoms in older adults^16^. The GAD-7 is a seven-item self-report questionnaire that evaluates the severity of generalised anxiety symptoms, widely used in clinical and research settings^17^. Table 5 presents the scoring scales for each assessment.

**Table 5:** Scoring scales for cognitive and mental health assessments.

Assessment	Scoring Range	Interpretation
Addenbrooke’s Cognitive Examination-III (ACE-III)	0 – 100	Higher scores indicate better cognitive function. A score below 82 suggests cognitive impairment.
Patient Health Questionnaire-9 (PHQ-9)	0 – 27	0-4: Minimal depression, 5–9: Mild, 10–14: Moderate, 15–19: Moderately severe, 20–27: Severe depression.
Geriatric Depression Scale-15 (GDS-15)	0 – 15	0–4: Normal (no depression), 5–8: Mild depression, 9–11: Moderate depression, 12–15: Severe depression.
Generalized Anxiety Disorder-7 (GAD-7)	0 – 21	0–4: Minimal anxiety, 5–9: Mild, 10–14: Moderate, 15–21: Severe anxiety.

### Dataset de-identification

A two-stage de-identification process was applied to the data. In the first stage, the data was pseudo-anonymised to develop analytical methods for the study. In the second stage, data was fully anonymised by removing all personally identifying information and any identifiable attributes. Participants are randomly assigned a Universally Unique Identifier (UID) to enhance security during de-identification. This ensures demographics and raw monitoring data from sleep mats and scan watches cannot be traced back to individuals while preserving the data’s utility for analysis.

Throughout the project, robust information governance, control methods, and procedures were implemented. An NHS-approved Data Processing and Impact Assessment was conducted to oversee the data collection, storage, and access procedures.

## Data Records

The **RESILIENT** dataset^10^ is publicly available on Zenodo and is organised into four main components: 1) A CSV file containing demographic information and baseline assessments related to mental health (PHQ-9, GAD-7, GDS-12) and cognitive functioning (ACE-III) for all participants. For ACE-III, both baseline and 6-month follow-up scores are included; 2) a metadata CSV file describing variables present in the demographic and devices data; 3) a CSV summary file providing per-participant data coverage statistics, including the number of recorded days, average records per day, and the earliest and latest timestamps. These metadata resources, as discussed in Table 3, enhance the dataset’s FAIRness by improving transparency and reusability; and 4) individual participant folders containing raw time-series data, including sleep states and physiological features captured by sleep mats, as well as step counts and heart rate data recorded by smart watches. More specifically, there are four tables included in each participant’s folder: ScanWatch Steps, ScanWatch HeartRate, Sleep States, and Sleep Physiology:

1. The ***ScanWatch Steps*** table records the number of steps detected by the watch within each one-hour interval, along with the corresponding timestamp.
2. The ***ScanWatch HeartRate*** table provides heart rate measurements taken every 10 minutes, along with their respective timestamps.
3. The ***Sleep States*** table records four sleep states (awake, light, deep, and REM) at given time intervals while the participant is in bed and positioned on the smart sleep mat.
4. The ***Sleep Physiology*** table contains physiological parameters such as heart rate, respiration rate, snoring, and heart rate variability. The latter is represented by the standard deviation of heart rate over a one-minute window (SDNN 1).
5. The ***Demographics*** table contains participant demographic information, including sex, age group, and four assessment scores used to evaluate depression, anxiety, and cognitive abilities: PHQ-9, GDS-15, GAD-7, and ACE-III. The tables include baseline scores for all assessments, with ACE-III also including scores from the 6-month follow-up. Additionally, two binary variables indicate whether a participant has been diagnosed with one or both of the two most common health conditions in the cohort: Essential Hypertension and Osteoarthritis.

Due to differences in sampling frequencies, these variables are recorded in separate tables. Each folder is named after the participant’s unique identifier (UID), allowing cross-referencing between the device data and the demographic information. Synchronisation between physiological measures and sleep states data is achieved using timestamp information provided by the Withings Sleep API, enabling temporal alignment across data despite differing data resolutions. Tables 1 and 2 provide an overview of the RESILIENT dataset^10^, detailing the structure and content of each data table, including recorded variables and their definitions, as well as a summary of recording coverage across data types in terms of average duration, sampling frequency, and recording span.

**Fig. 2:**
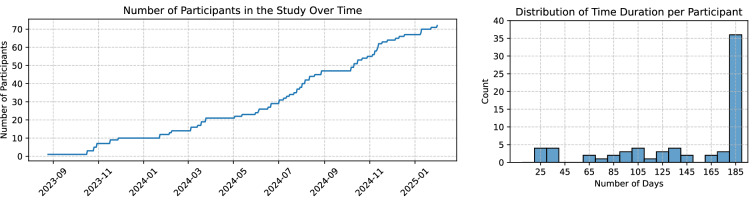
Overview of cumulative number of participants and their durations in the study.

**Fig. 3:**
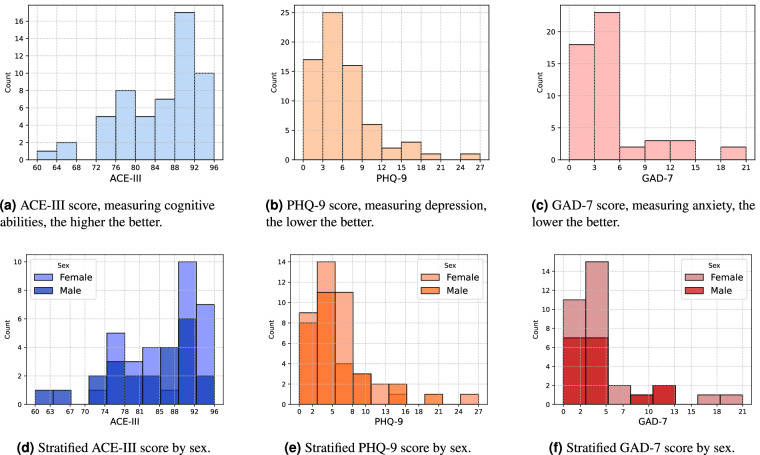
Overview of **baseline** assessment scores by histograms, including sex-stratified distributions.

Figure 2 displays the cumulative number of participants enrolled over time and the distribution of participation durations. Notably, the maximum duration for any participant is 185 days in this dataset. Figure 3 shows the histograms of three assessment scores included in the Demographics table (Table 4), ACE-III at baseline, PHQ-9, and GAD-7, along with their stratification by sex. These scores assess cognitive function, depression, and anxiety, respectively. The dataset also includes the GDS-15 score, another measure of depression, which displays a distribution similar to that of the PHQ-9.

## Technical Validation

To validate the technical quality of the data, we conducted several analyses: a subgroup comparison across sex and age groups, a correlation analysis between cognitive and mental health assessment scores and signals from sleep mats and smart watches stratified by sex, and a set of visualisations to evaluate the alignment of participants’ signal trajectories.

**Fig. 4:**
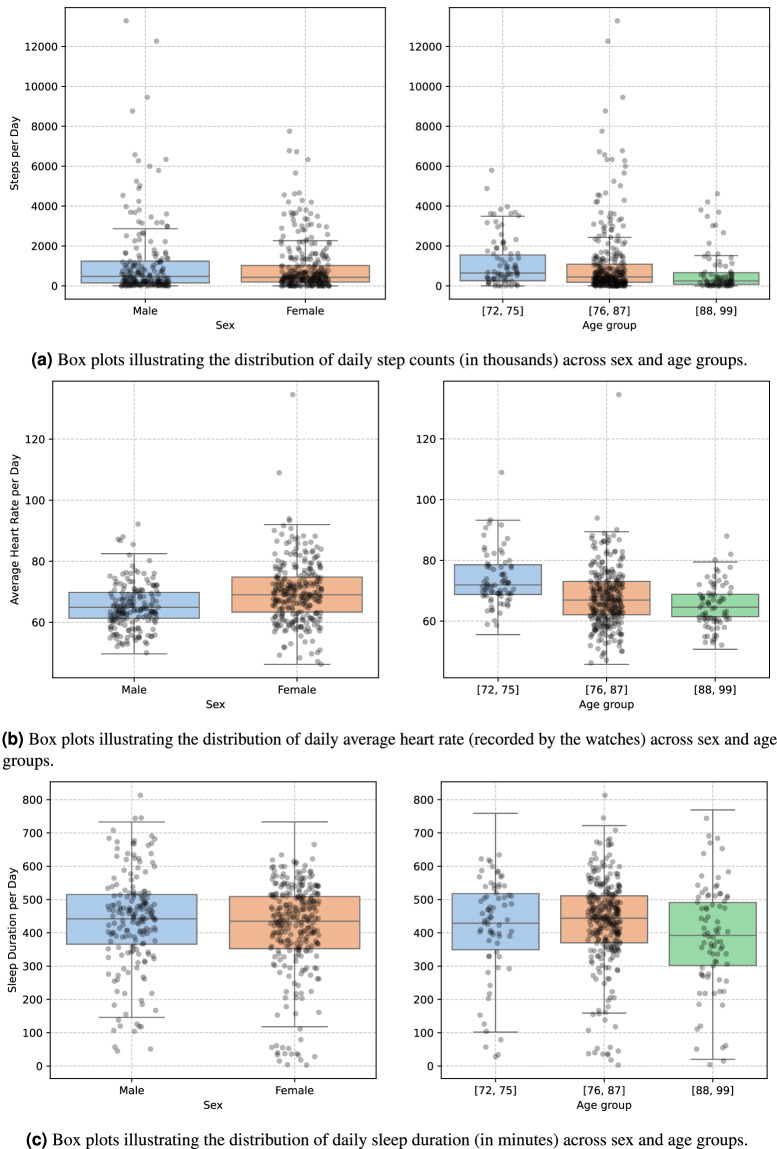
Comparing the distributions of daily steps, sleep duration, and average heart rate between sex and age groups.

Figure 4a–c present the results of the subgroup analysis comparing daily step count, sleep duration, and average heart rate between sex and age groups. The box plots reveal that females have a higher average heart rate (69.89 bpm ± 9.35) compared to males 65.91 bpm ± 7.49), while daily step counts are lower among females (881.66 steps ± 1266.85) than males (1167.70 steps ± 1915.93). Sleep duration is also slightly shorter in females (415.34 minutes ± 35.27) than in males (435.23 minutes ± 138.61). These patterns align with previous findings in the literature, which report sex-based physiological differences in metrics such as heart rate and activity levels^18^.

Figure 4 also illustrates the distributions of daily step count, average heart rate, and sleep duration across different age groups represented in the dataset. Figure 4a shows a decline in daily step count with increasing age. The youngest age group (72–75) displays the highest median and the widest distribution, while the oldest group (88–99) has the lowest median and a more concentrated range. This pattern supports prior findings that physical activity tends to decline with age due to reduced mobility, lower energy levels, and age-related health conditions^19,20^.

Figure 4b reveals a similar age-related trend in heart rate. The youngest group exhibits the highest median heart rate, while the oldest group records the lowest. This decline probably corresponds to the reduction in physical activity and is consistent with previous research indicating a gradual decrease in heart rate within the ageing process^21^. In contrast, Fig. 4c shows that average daily sleep duration remains relatively stable across age groups, with no substantial variation observed.

**Fig. 5:**
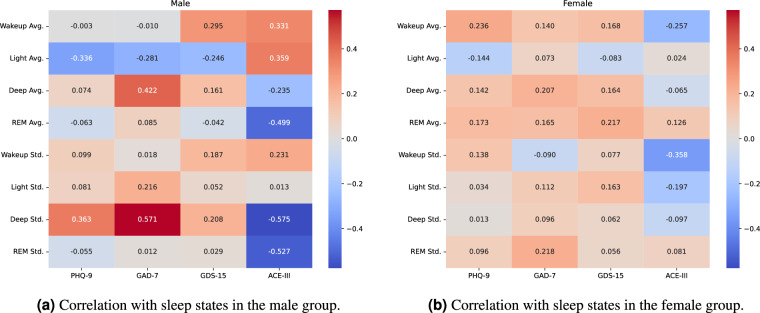
Heatmaps showing correlations between **baseline** mental health assessments and sleep measures recorded by the sleep mat over a 7-day period following the assessment date: (**a**) sleep states in males, (**b**) sleep states in females.

**Fig. 6:**
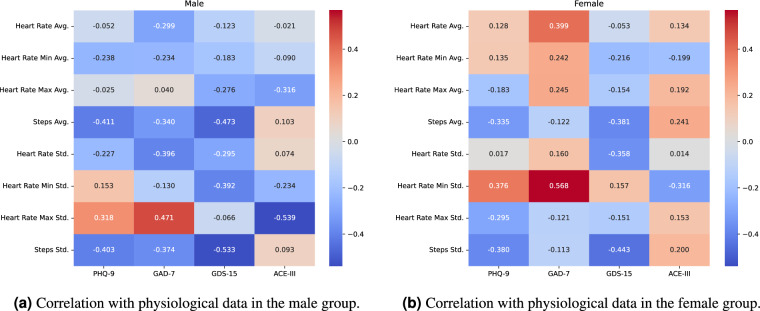
Heatmaps showing correlations between **baseline** mental health assessments and physiological measures recorded by the watch over a 7-day period following the assessment date: (**a**) heart rate and step counts in males, (**b**) heart rate and step counts in females.

Figures 5 and 6 present correlation heatmaps between cognitive and mental health assessments at baseline and both sleep states and watch, derived physiological signals, stratified by sex. The data reflect daily averages and standard deviations calculated over a seven-day period following each assessment. No significance testing or inferential modelling was performed, and these analyses are intended to explore patterns that may warrant further investigation.

Among males (Fig. 5a), we observed moderate to high correlations between increased variability in deep sleep and higher anxiety (GAD-7: r = 0.571) and depression scores (PHQ-9: r = 0.363). Similarly, lower ACE-III baseline scores were associated with greater variability in REM (r = –0.527) and deep sleep (r = –0.575), and with shorter REM duration (r = –0.499). These descriptive findings are consistent with literature suggesting links between sleep stability and cognitive and mental health^22,23^.

In females (Fig. 5b), the correlations were generally weaker. Notable descriptive associations included REM variability with anxiety (GAD-7: r = 0.218), REM duration with depression (GDS-15: r = 0.217), and wake-up time variability with cognition (ACE-III baseline: r = –0.358). These sex differences may reflect initially underlying physiological or behavioural factors affecting female participants^24^.

For physiological signals derived from the scan watch (Fig. 6a,b), we found that in females, higher average and minimum heart rate correlated with anxiety (GAD-7: r = 0.399 and r = 0.242, respectively), and to a lesser extent, depression (PHQ-9: r = 0.128). In contrast, in males, maximum heart rate variability showed moderate associations with higher anxiety (GAD-7: r = 0.471) and depression (PHQ-9: r = 0.318), and lower cognitive performance (ACE-III baseline: r = –0.539). These patterns align with prior observational research on heart rate variability and mental health^25,26^.

Physical activity was also descriptively associated with mental health outcomes. Lower and less variable step counts correlated with higher depressive symptoms in both sexes, aligning with previous findings on sedentary behaviour and mental health^27^. The correlation between step count variability and depression (GDS-15) appeared stronger in males (r = –0.533) than in females (r = –0.443), although these are descriptive patterns without statistical testing.

**Fig. 7:**
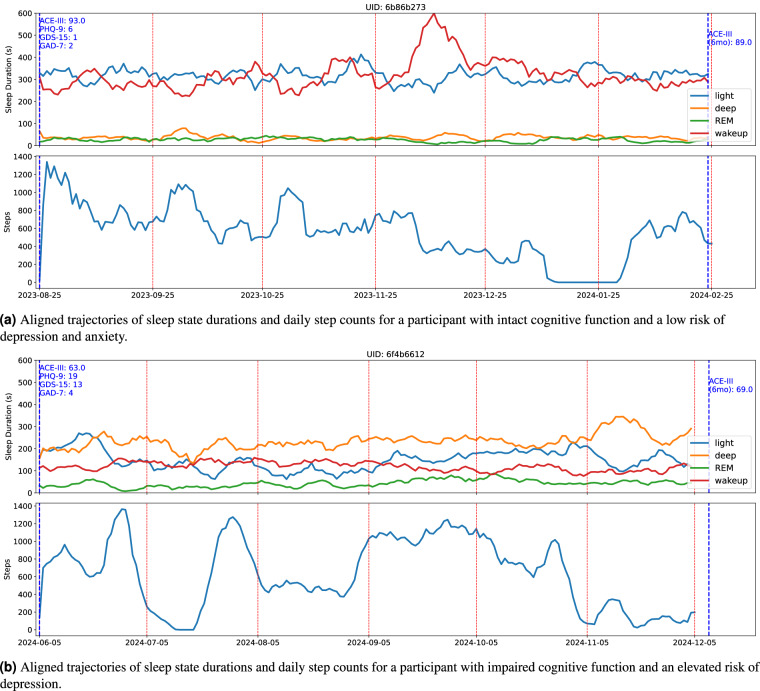
Aligned trajectories of sleep state durations and daily step counts for two participants with contrasting profiles: one with intact cognitive function and low risk of depression and anxiety, and the other with impaired cognitive function and an elevated risk of depression. Baseline assessments were completed for all mental health measures, with a 6-month follow-up for the ACE-III.

Figure 7a illustrates a participant with intact cognitive function and low risk of depression and anxiety, as indicated by a high ACE-III score at baseline and low PHQ-9 and GDS-15 scores. This participant shows relatively consistent sleep architecture over time, with light sleep and wake durations being most dominant. Their step count trajectory also shows regular patterns, and periods of reduced physical activity are sometimes mirrored by increased wake durations, suggesting a potential relationship between activity and sleep quality. Cognitive performance, as measured by the ACE-III, declined slightly but remained within the range of healthy cognitive function at the 6-month follow-up. In contrast, Fig. 7b depicts a participant with impaired cognitive function and a higher risk of depression, as indicated by a low ACE-III at baseline score and high PHQ-9 and GDS-15 scores. This participant shows longer durations of deep sleep, and the step counts do not display a clear correlation with sleep states. Similar differences in sleep patterns between individuals with and without cognitive impairment have been observed in previous studies^28^. At the 6-month follow-up, the ACE-III score shows a slight improvement but remains within the range indicative of cognitive decline, aligning with increased deep sleep duration, reduced light sleep, and decreased physical activity.

## Usage Notes

Designed to advance clinically relevant machine intelligence and decision-support systems, the RESILIENT dataset^10^ combines longitudinal data with assessments of mental health and cognitive function, facilitating continuous healthcare monitoring. In addition to the raw data, we have open-sourced the software platform^9^ used for collecting data from smart devices. We also provide comprehensive guidelines^10^ for accessing, visualising, and analysing correlations between sleep states, physical activity, and mental health.

The dataset is organised as one demographic table for all participants and individual folders for each participant, including four separate tables stored as separate Comma-separated values (CSV) files: sleep states, sleep physiology, steps and heart rate recorded by watches. Data can be cross-referenced across the files. The instructions for loading the data and a set of sample codes for aggregating and analysing the dataset are also provided in the GitHub repository https://github.com/tmi-lab/Resilient-Dataset.

## Data Availability

The RESILIENT dataset is publicly available via its dedicated Zenodo repository^10^. To support reproducibility and further exploration, we provide two complementary open-source codebases: • Data analysis and visualisation code: Available at https://github.com/tmi-lab/Resilient-Dataset, this repository contains the code used to generate all summary statistics, figures, and stratified analyses (e.g., by sex and age group) presented in this document. It includes a detailed README file describing the structure of the data records, along with a “*requirements.txt*” file listing all software dependencies and versions. • RESILIENT platform code: Available at https://github.com/tmi-lab/resilient, this repository provides the source code for the full RESILIENT platform used to collect, process, and manage in-home monitoring data. This includes tools for wearable data acquisition, data pre-processing, storage infrastructure and report generation.
